# Identification of candidate mediators of chemoresponse in breast cancer through therapy-driven selection of somatic variants

**DOI:** 10.1007/s10549-020-05836-7

**Published:** 2020-07-30

**Authors:** Waleed S. Al Amri, Diana E. Baxter, Andrew M. Hanby, Lucy F. Stead, Eldo T. Verghese, James L. Thorne, Thomas A. Hughes

**Affiliations:** 1 School of Medicine, University of Leeds, Leeds, UK; 2 Department of Histopathology and Cytopathology, The Royal Hospital, Muscat, Oman; 3 Department of Histopathology, St. James’s University Hospital, Leeds, UK; 4 School of Food Science and Nutrition, University of Leeds, Leeds, UK

**Keywords:** Chemoresistance, Exome sequencing, Somatic variants, Sensitization

## Abstract

**Purpose:**

More than a third of primary breast cancer patients are treated with cytotoxic chemotherapy, typically without guidance from predictive markers. Increased use of neoadjuvant chemotherapy provides opportunities for identification of molecules associated with treatment response, by comparing matched tumour samples before and after therapy. Our hypothesis was that somatic variants of increased prevalence after therapy promote resistance, while variants with reduced prevalence cause sensitivity.

**Methods:**

We performed systematic analyses of matched pairs of cancer exomes from primary oestrogen receptor-positive/HER2-negative breast cancers (*n* = 6) treated with neoadjuvant epirubicin/cyclophosphamide. We identified candidate genes as mediators of chemotherapy response by consistent subclonal changes in somatic variant prevalence through therapy, predicted variant impact on gene function, and enrichment of specific functional pathways. Influence of candidate genes on breast cancer outcome was tested using publicly available breast cancer expression data (*n* = 1903).

**Results:**

We identified 14 genes as the strongest candidate mediators of chemoresponse: TCHH, MUC17, ARAP2, FLG2, ABL1, CENPF, COL6A3, DMBT1, ITGA7, PLXNA1, S100PBP, SYNE1, ZFHX4, and CACNA1C. Genes contained somatic variants showing prevalence changes in up to 4 patients, with up to 3 being predicted as damaging. Genes coding for extra-cellular matrix components or related signalling pathways were significantly over-represented among variants showing prevalence changes. Expression of 5 genes (TCHH, ABL1, CENPF, S100PBP, and ZFHX4) was significantly associated with patient survival.

**Conclusions:**

Genomic analysis of paired pre- and post-therapy samples resulting from neoadjuvant therapy provides a powerful method for identification of mediators of response. Genes we identified should be assessed as predictive markers or targets in chemo-sensitization.

**Electronic supplementary material:**

The online version of this article (10.1007/s10549-020-05836-7) contains supplementary material, which is available to authorized users.

## Introduction

Primary solid cancers are increasingly commonly treated with systemic therapies *before* resection surgery, referred to as neoadjuvant systemic therapies, rather than the more traditional approach of surgery first [1, 2]. This is because neoadjuvant protocols present clinical advantages, while still achieving the key aim of targeting disseminated disease. Advantages include that primary tumours can be reduced in size, potentially allowing less radical surgical resection [3], and response to therapy can be monitored by imaging of the primary tumour, potentially allowing regimens to be modified if responses are inadequate [4]. A consequence of the increased use of neoadjuvant protocols is the availability for research purposes of matched samples of primary tumour tissue taken before systemic therapy, usually in the form of a diagnostic biopsy, and after therapy from the resection. These matched samples present a powerful resource for study of the molecular response of tumours to therapy, and thereby to identify pathways associated with relative therapy resistance or sensitivity [5–7]. The hypothesis behind such analyses is that tumour cells that remain after therapy include characteristics associated with therapy resistance, while characteristics present before therapy but lost in the matched post-treatment sample include molecular events associated with relative therapy sensitivity.

With respect to cancer genomics, this hypothesis implies that somatic variants that expand in prevalence after therapy may promote resistance, while somatic variants that are eliminated or reduced in prevalence may be associated with therapy sensitivity [6]. However, such studies are in their infancy for solid cancers as they are limited by technical and analytical challenges relating to the small size of samples, whether these samples are representative of the diversity of somatic clones throughout the tumour, whether variable infiltration with non-cancer (stromal) cells invalidates assessment of variant prevalence, and the difficulty of separating functionally response-modifying variants from passengers. For example, in the context of breast cancer and cytotoxic chemotherapy, one of the most common neoadjuvant systemic therapy settings, we are aware of only five such studies [6, 8–11].

We have recently performed a study into the genomic selection cytotoxic chemotherapy exerts on primary breast cancers using this matched pre- and post-therapy design [6]. Importantly, we used laser microdissection to select tumour cells from samples before sequencing whole cancer exomes, in order to allow comparison of the prevalence of somatic variants without the confounder of variable stromal contamination. We successfully identified two genes, MUC17 and PCNX1, that hosted somatic variants showing evidence of selection by therapy and subsequently validated their potential as mediators of therapy response. Here, we present a prioritized list of candidate mediators of chemoresponse in breast cancer, which was identified from our novel dataset using a systematic pipeline for analysis of these paired cancer genomes. We also confirm that the levels of expression of these genes impact on breast cancer outcomes using publicly available expression data, thereby validating our selection methodology and identifying genes of importance for future functional analyses.

## Methods and materials

### Patient recruitment and data acquisition

This has already been described in detail [6], a brief summary follows. Ethical approval for this work was obtained from Leeds (East) REC (ref. 06/Q1206/180). Patients gave informed, written consent for use of their tissues in accordance with this permission, and the study protocol conformed to the Declaration of Helsinki. Data are reported in accordance with REMARK [12] where appropriate. 6 patients undergoing neoadjuvant chemotherapy using epirubicin/cyclophosphamide for primary oestrogen receptor-positive/HER2-negative breast cancer at the Leeds Teaching Hospitals NHS Trust and showing only partial responses were included in the study. Tumour samples from before chemotherapy (diagnostic biopsies) and after chemotherapy (resection) were available. Tumour cells were isolated by laser capture microscopy using a Zeiss/PALM machine (Zeiss, Oberkochen, Germany). Whole-exome sequencing data were obtained from tumour cells, and from matched normal tissues (normal tissue adjacent to the tumour) using SureSelectXT reagents (Agilent Technologies) and the HiSeq 3000 (Illumina), with paired-end reads (150 bp). Sequence data have been deposited at the European Genome-phenome Archive, under accession number EGAS00001003626 (https://ega-archive.org).

### Sequencing analysis, variant calling and intra- and inter-patient comparisons

Whole-exome sequencing data were processed as previously described [6] using open-source bioinformatics tools by Edinburgh Genomics Laboratory (Edinburgh, UK). In summary, adapters, primers and poor-quality bases were trimmed using cutadapt (v1.8.3) [13] and trimmed reads were aligned to reference genome Hg19 using BWA-MEM (v0.7.15) [14]. PCR duplicates were marked using the MarkDuplicates tool from the Picard tools package (v1.115). Base quality score recalibration (BQSR) was performed using BaseRecalibrator from GATK (v3.7) [15]. MuTect2 (GATK v3.7) was used to detect somatic variants, and the HaplotypeCaller pipeline (GATK v3.7) was used to detect germ-line variants. Variant filtering was performed using SelectVariants from GATK, to exclude variants with a read depth of less than 5 or more than 800, or a quality Phred score of less than 30. Variant metrics were extracted using snpSift tool extractFields [16]. Changes in mutant allele frequency (MAF) were determined when the exact same variant was detected and survived filtering both pre- and post-therapy in the same case—a threshold of changes > 5% was set for inclusion in subsequent analyses. Variants were defined as unique to pre- or post-therapy when this variant was not detected or did not survive filtering in the matched sample.

### In silico analyses

The ToppGene suite was used for functional enrichment analyses [17]. Expression data were accessed through cBioPortal [18]. METABRIC [19] data were selected from the section labelled breast, under the subcategory Invasive Breast Carcinoma. mRNA expression *z*-scores was highlighted, and mutations and copy number were deselected. Genes of interest were submitted and expression downloaded in a tab delimited format and analysed in Prism v8 (Graphpad, San Diego, USA).

## Results

### Somatic variants were best defined by comparison to pooled normal sequences

Our first aim was to assess different ways of defining somatic (cancer-specific) genomic variants in each of the separate pre- and post-therapy samples. Variants within the sequencing data of either cancer or normal samples were considered for further analysis if their read depths were greater or equal to 5 and less than 800, and if they had quality Phred scores of greater than or equal to 30. Next, somatic variants were identified as variants seen in the cancer samples but absent in the *matched* normal sample—resulting in a mean somatic variant burden of 633 (range 72–2719; see Table 1).

**Table 1:** Somatic variants were more stringently defined by comparison to pooled germ-line sequences than to matched individual germ-lines

Sample	Somatic variants (not in matched normal)				Somatic variants (not in any normal)			
	SNV	Ins	Del	All	SNV	Ins	Del	All
1: pre-NAC	174	9	14	197	68	7	5	80
1: post-NAC	80	6	7	93	36	3	6	45
2: pre-NAC	2585	53	81	2719	1355	25	54	1434
2: post-NAC	58	6	8	72	43	4	6	53
3: pre-NAC	228	80	91	399	124	76	87	287
3: post-NAC	385	60	20	465	112	54	14	180
4: pre-NAC	401	67	89	557	339	62	85	376
4: post-NAC	439	47	47	533	238	42	44	324
5: pre-NAC	952	154	83	1189	125	135	70	330
5: post-NAC	931	38	48	1017	137	26	33	196
6: pre-NAC	102	36	25	163	38	33	23	94
6: post-NAC	133	28	26	187	42	21	24	87
Mean % change using all normals					− 56%	− 22%	− 20%	− 50%

Somatic variants in cancer cells (either pre- or post-NAC) were identified from six breast cancers from exome sequencing data by comparison to sequencing of the individual patient-matched normal genome (left columns), or by comparison to the pooled variants from all six normal genomes (right columns). Total numbers of variants are shown (All), as well as broken down as single-nucleotide variants (SNV), insertions (Ins), and deletions (Del). The mean % difference in variant count between use of matched or all normals is shown in the bottom row

It should be noted that 5 or more reads of a variant in a cancer sample represents adequate proof of the presence of that variant, but absence of a variant in the normal sequence is harder to prove since heterozygous variants can be missed by chance depending on read depth [20]. Median read depths of our normal samples in this study ranged from 15 to 46. As an example, at this lowest median depth, probability calculations defined that ~ 0.003% of heterozygous germ-line variants would be called as homozygous wild type in error. Since the typical ‘normal’ genome varies from the reference genome at between 4.1 and 5 million positions [21], this represents a risk of misidentifying hundreds of variants. In order to mitigate this, we also identified somatic variants by comparing each cancer sample to the pooled variants identified in all 6 normal samples—effectively pooling the read depths thereby reducing the probability of missing heterozygote germ-line variants, which scales 2^*n*^ with read depth, to negligible. This resulted in a mean somatic variant burden of 291 (range 45–1434; substantially lower than previously; Table 1). Interestingly, single-nucleotide variants (SNV) were much more commonly filtered out by this strategy than either insertions or deletions (Table 1), suggesting that more SNV were missed in the germ-line sequencing than the other aberrations. We believe the use of pooled normal variants allows more robust identification of somatic mutations when read depth is limiting [20], at the cost of potentially losing true somatic variants from individual cases that exactly match germ-line variants from another case. This match between somatic and germ-line variants would normally be regarded as very unlikely; however, it could be argued that this risk is greater where the cohort was assembled on the basis of sharing a tumour phenotype (in this case, relatively poor response to chemotherapy) to which either somatic or germ-line variants could contribute [22]. Nevertheless, the benefits of greatly reducing mis-calling of somatic variants outweigh the risk of missing rare true positives.

### Comparisons between pre- and post-NAC samples identified candidate mediators of resistance and sensitivity

Next, in order to identify variants that changed in allelic frequency during chemotherapy indicating a potential role in defining relative resistance or sensitivity, we compared the somatic variant profiles in the matched pre- and post-NAC samples. Variants were assigned to the two following groups: (A) unique to the pre-NAC sample, or reduced in mutant allele frequency (MAF) after therapy if detected both pre- and post-NAC (i.e. lost or reduced in prevalence after NAC) and (B) unique to the post-NAC sample, or increased in MAF after therapy if detected in both samples (i.e. selected for by NAC). The numbers of variants in these two groups for the 6 cases ranged from 73 to 1488 for group A and 35 to 232 for group B, representing pooled totals of 2488 variants for list A and 751 variants for list B; these variants are listed in Table S1.

### Prioritization based on comparisons between cases

Our next aim was to prioritize these potential mediators of chemotherapy response for future downstream analyses, by estimating their likelihoods of being true mediators of response as opposed to passenger variants. Our first strategy was to look for commonalities between the 6 different cancer cases. There were no somatic variants in common between the different cancers, which reflect the huge genetic heterogeneity of breast cancers [23]. However, many somatic variants were identified within the same gene in different cancers.

112 genes hosted variants that were categorized into list A in 2 or more cancers (100 genes had variants in two cases, 11 in three, and 1 in four). 21 genes hosted variants categorized into list B in 2 or more cancers (20 genes represented in two cases, and 1 gene in three cases). These 131 genes (not 133 genes: see below), termed list C, are potentially enriched for genes having an impact on chemoresponse, making the assumption that the different variants in each gene were functionally similar driving the same change in response (ie all loss-of-function or all gain-of-function). Note that two genes (MUC17 and ZDHHC11) were categorized onto list C twice, through variants for which the allelic frequency decreased through therapy in multiple patients *and* through different variants being consistently increased in allelic frequency through therapy (from list A, both genes had variants in three cases and from list B, both genes had different variants in two cases). If these variants all contribute to therapy response, it implies that for each gene, one set of variants must be loss-of-function while the other set must be gain-of-function. There are few individual genes within the literature that undergo both loss-of-function and gain-of-function somatic cancer mutations, with GATA3 [24] and p53 [25] providing examples. This was considered unlikely in the case of these two genes, although both genes may be functionally relevant in one of the loss-of-function or gain-of-function settings and therefore remain as candidate mediators of response.

It is also worth highlighting that a further 126 genes had at least one variant on list A and one variant on list B, again implying both loss-of-function and gain-of-function variants if these variants both contribute to therapy response. Most surprisingly, for 17 of these genes, variants from list A and list B were identified in the same individual patients. Our interpretation is that because these examples do not show consistent directions of frequency alteration through therapy across different patients, they should not be prioritized. The risk with this strategy is abandoning a rare and scientifically interesting example of loss-of-function and gain-of-function variants within single genes, hidden within these probable false positives.

### Prioritization based on functional enrichment analyses

The lists of genes identified so far were, at best, enriched for genes potentially involved in chemoresponse, on the basis of changes in variant frequency in individual (lists A and B) or multiple cancers (list C). A further method of identifying functionally relevant genes within these lists was to search for any over-represented molecular functions, based on the hypothesis that variants in a number of different genes might be responsible in different individuals for deregulation of the same molecular pathway that defines chemoresponse. Genes within over-represented pathways would represent stronger candidate mediators, although a risk with this strategy is the potential to identify pathways that require multiple aberrations to exert a strong functional influence and therefore the impact of each individual gene is challenging to validate in downstream functional analyses.

We performed gene set enrichment analyses on gene lists A, B, and C (Table S2). A variety of molecular pathways were significantly over-represented among the mutated genes in each category. Of particular note were the pathways consistently identified in all three lists, which may indicate potential deregulation by loss-of-function and gain-of-function variants in a wide range of individual genes; these were extra-cellular matrix, glycoproteins, collagens and proteoglycans; integrin signalling pathway molecules; and, structural components of basement membranes.

### Prioritization based on predictions of functional impact of variants

A variety of well-established bioinformatics tools are available to predict the impact of individual variants on gene function [26]. Variants predicted to have a potent impact on gene function would be less likely to be passenger mutations, and would therefore be stronger candidate mediators of chemoresponse. We used these tools on the variants identified in list C, which we expect to be enriched in true positive (functional) variants by previous analyses, and compared these outcomes to those from the variants on lists A and B, and the entire initial list of somatic variants. Of the 336 variants that occurred within the 131 genes of list C, 82.1% were predicted to be damaging by at least one of SIFT (Sorting Intolerant from Tolerant, scores below 0.05) or PolyPhen2. 42.1% of variants from the pooled list A and B were predicted to be damaging, while this value was 53.6% for the entire list of somatic variants. The fact that list C is heavily enriched for damaging variants, as compared to the longer lists from which the genes on list C were selected, supports our prioritization strategy based on both changes in allelic frequency through therapy and commonalities between patients, increasing the justification for further examination of the individual genes with damaging variants with respect to chemoresponse.

### Final prioritization of candidate genes

Next, we integrated the pathway enrichment and the predictions of variant effect analyses to produce a prioritized list of candidate genes as mediators of chemotherapy response. Prioritization order of genes was initially defined by the number patients in which variants showing selection were found (2, 3 or 4; more patients giving higher priority). This was further ordered based on the number of these variants that were predicted to be damaging (potentially up to 7 since some patients showed a change in prevalence of multiple variants within individual genes; larger number giving higher priority). Finally, the list was ordered on whether genes were listed in any of the top 3 enriched pathways from lists A, B or C. This process of prioritizing resulted in an ordered list of the 131 candidate genes (with MUC17 and ZDHHC11 each having two separate entries representing variants increased or decreased in prevalence). We set an arbitrary threshold for scores of 5 or greater (number of patients + number of damaging variants + 1 if in an enriched pathway) to give 14 genes in our highest priority category as having the strongest potential to be mediators of chemoresponse in breast cancer (Table 2 shows the genes and their prioritization scores; Table S3 shows the variants leading to their selection). The steps taken to reach this final list of candidate genes are illustrated in a flow chart (Fig. S1).

**Table 2:** Prioritized list of genes showing the strongest evidence of involvement in defining chemoresponse to epirubicin/cyclophosphamide in breast cancer

Gene	Selected against (A) or for (B)?	No of tumours?	Damaging predictions?	Pathway (yes/no)?	Priority total
TCHH	A	3	3	N	6
MUC17	B	2	3	N	5
ARAP2	A	3	2	N	5
FLG2	B	3	2	N	5
ABL1	A	3	2	N	5
CENPF	A	2	3	N	5
COL6A3	A	2	2	Y; collagen proteins	5
DMBT1	A	4	1	N	5
ITGA7	A	2	2	Y; integrin signalling pathway	5
PLXNA1	A	2	3	N	5
S100PBP	A	2	3	N	5
SYNE1	A	3	2	N	5
ZFHX4	A	2	3	N	5
CACNA1C	B	2	2	Y; type II diabetes mellitus	5

Genes were identified that hosted somatic variants showing selection by therapy. Genes were prioritized on the basis of how many cases showed a consistent direction of selection (column 3), how many variants were predicted to be damaging (column 4), and whether the gene functions in a pathway that was over-represented in the lists of genes showing selection (column 5). These factors were combined (column 3 + column 4 + 1 if Y in column 5) to give a final prioritization score (column 6)

### Expressions correlated with breast cancer outcomes

**Fig. 1:**
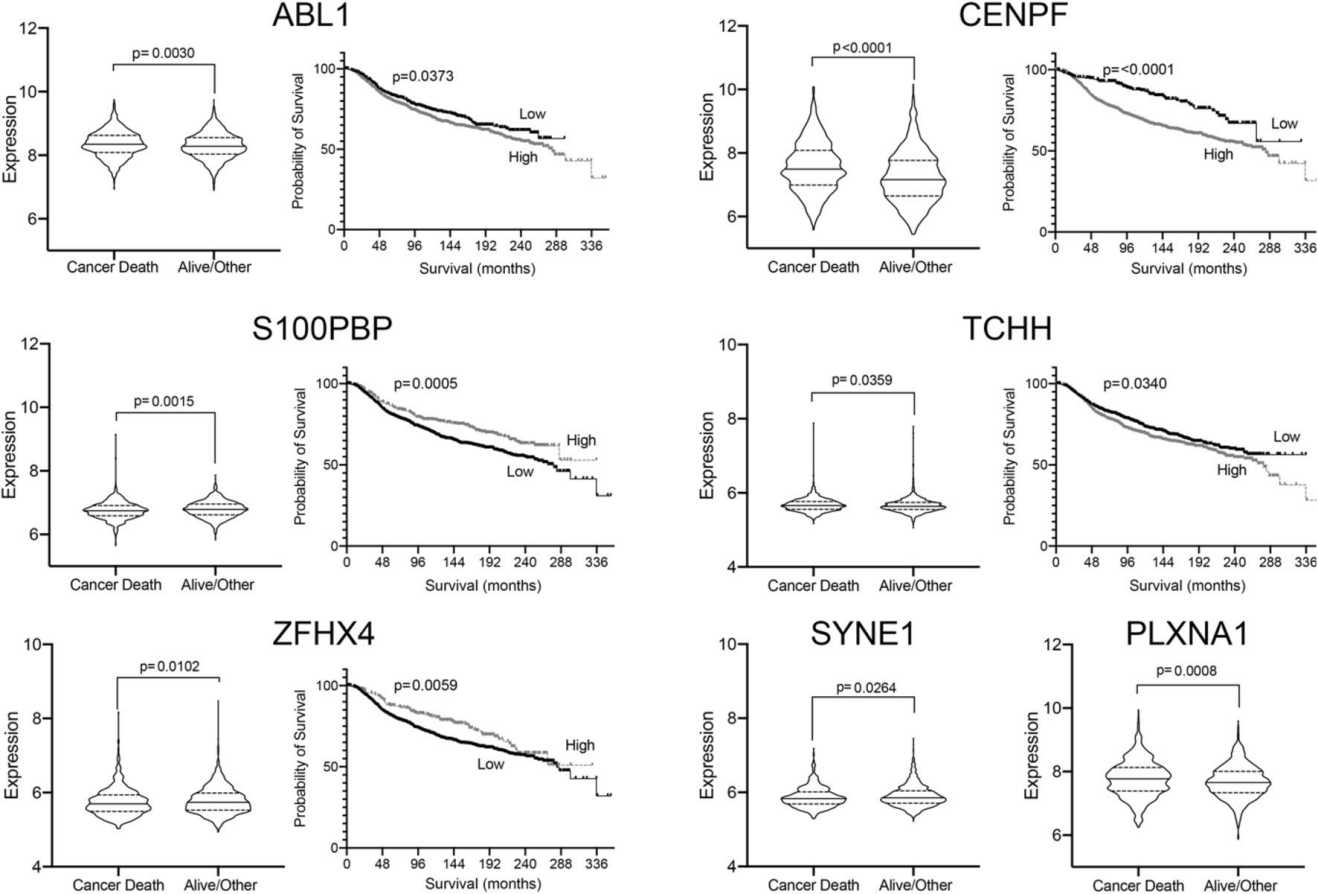
Expression of candidate genes correlated with breast cancer outcomes. Expression levels of candidate genes in Table 2 were analysed for correlations with survival from breast cancer using the METABRIC dataset [19], by comparing the distribution of levels between patients who died of their cancer to those that did not using ‘violin’ plots (left of each pair), and by Kaplan–Meier analyses after expression was dichotomized using receiver operator curve analyses into low and high groups (right of each pair). For violin plots, median and quartiles are shown (horizontal lines) and significance was tested using 2-tailed Mann–Whitney *U* tests. For Kaplan–Meier analyses, significance was tested using log rank tests. Significant correlations only are shown; PLXNA1 and SYNE1 were significant in only the first analysis

Finally, to support the potential importance of our 14 highest priority genes in defining breast cancer chemoresponse and patient survival, we assessed whether expression levels correlated with cancer outcomes using publicly available transcriptome data for primary breast cancer samples. Using the METABRIC dataset (*n* = 1903) [19], we tested whether expression of each individual gene was associated with survival status (ie died from breast cancer vs alive/lost to follow up) by receiver operator curve analyses. 7 genes (TCHH, ABL1, CENPF, PLXNA1, S100PBP, SYNE1, ZFHX4) were significantly associated (individual *p* < 0.05, and multiple test corrected FDR < 5%). All of these genes showed a significant difference in the distribution of expression levels between the groups that died from breast cancer (*n* = 622) vs alive/lost to follow--up (*n* = 1281) (*p* < 0.05) (see ‘violin’ plots in Fig. 1). In addition, 5 of these significantly predicted differences in length of survival using Kaplan–Meier survival analyses (*p* < 0.05; right plot of the pairs, Fig. 1). Since our initial cohort used for genomic sequencing included only oestrogen receptor-positive/HER2-negative cases, we also analysed only these cases from the METABRIC dataset (*n* = 1350). This analysis is potentially more relevant since it matches our initial observations in terms of molecular cancer subtype; however, it could be argued it is also less relevant since a smaller proportion of these cases would have been treated with chemotherapy in the primary setting. In this analysis, expression of 2 genes (CENPF, PLXNA1) showed significant differences between groups that died from breast cancer (*n* = 388) vs alive/lost to follow up (*n* = 962) (*p* < 0.05), and significantly predicted differences in length of survival in Kaplan–Meier analyses (*p* < 0.05) (Fig. S2). We concluded that our analysis has successfully identified genes that impact on breast cancer outcomes.

## Discussion

Cytotoxic chemotherapy has been used in primary breast cancer treatment for more than 60 years [27], yet patients are still stratified to the therapy without molecular insights into whether their individual cancers will respond. Almost all metastatic breast cancer patients receive cytotoxic chemotherapy, and eventual failure to respond leads to patient death. In both primary and metastatic settings, activity of molecular pathways and expression of individual genes that define tumour responses are poorly understood. We present a strategy for identification and prioritization of candidate mediators of chemoresponse using paired cancer exome data taken pre- and post-therapy. We use this strategy to identify molecular pathways involved with defining responses in breast cancer (Table S2), as well as 14 high-priority specific candidate genes (Table 2). The relatively small size of our cohort (*n* = 6) is a limitation for our study. However, it is worth noting that this is in the same range as the only two other studies to sequence genomes of matched pre- and post-chemotherapy primary breast cancer samples (*n* = 20 [9], *n* = 9 [11], and our work is the only study to employ laser capture of cancer cells to allow robust comparisons of mutant allele frequencies.

We have already validated the role of one of these genes, MUC17, using in vitro approaches and further cohorts of chemotherapy-treated patients [6]. Here, we assessed whether expression levels of candidate genes correlated with breast cancer outcomes using publicly available data from the METABRIC study [19]. Although 7 of the genes (50%) showed significant correlation with outcome, thereby supporting our priority gene list, it is worth emphasizing the issues with this approach and therefore that the genes failing to show significant correlations remain candidate chemoresponse mediators. Most obviously, our initial exome data focused on cancer cells only (isolated by laser microdissection), while the METABRIC expression data include contributions of variable amounts of stroma [19] that could mask true relationships for the cancer cells. In addition, the patients within METABRIC received a wide range of treatments for their primary disease [19], not always including cytotoxic chemotherapy, this issue is mitigated to some extent by our use of cancer-specific survival as the end-point, since almost all patients will have received cytotoxic chemotherapy for metastases, but we are unable to assess whether metastatic expression was concordant with the analysed levels in primaries. Two of the genes that were validated in the METABRIC data have previously been reported as mediators of breast cancer chemotherapy response. ABL1, a non-receptor tyrosine kinase, has been implicated in response to DNA-damaging chemotherapeutics in tissue culture [28], while high expression of the centrosomal protein CENPF has been associated with good chemoresponses in breast cancers [29]. Of the other validated genes, only one has previously been associated with cytotoxic chemotherapy response in other cancers: somatic variants in SYNE1, which codes for a nuclear envelope-associated protein [30], correlated with poor response to induction chemotherapy in head and neck cancer [31]. The remaining four genes have diverse cancer-related associations. For example, over-expression of TCHH, which encodes trichohyalin, a structural protein that binds keratin fibres [32], has been linked with sensitivity to tyrosine kinase inhibition in bladder cancer [33]. PLXNA1, a semaphorin receptor, has a range of influences that can be either pro- [34] or anti-tumourigenic [35]. S100PBP expression correlated with spread to different metastatic sites in breast cancer [36], although its precise molecular function is poorly understood. The transcription factor ZFHX4 has been reported as required for maintenance of tumour-initiating cells in glioblastoma [37].

With respect to molecular pathways (Table S2), we implicate extra-cellular matrix (ECM) components, including collagens and laminins, and signalling molecules that interact with the ECM, including integrins, in chemoresponse (Table S2). Associations between ECM and therapy response are well reported [38, 39],a prevailing model suggests that relatively dense ECM presents a physical barrier restricting drug movement, and thereby mediating cancer cell survival through reduced local concentrations, although some more specific molecular signalling is also implied [38]. Two genes from these pathways were included in our final prioritized list: COL6A3 and ITGA7. COL6A3 itself has previously been implicated in mediation of chemoresistance in breast cancer, with upregulation of collagen VI, the heterotrimer to which COL6A3 contributes, in breast cancers [40], and endotrophin, a soluble C-terminal domain cleaved from the COL6A3 protein, associated with cisplatin resistance in a mouse model [41]. ITGA7, which acts as a receptor for a number of laminins [42, 43], has also recently been implicated in chemoresponse [44, 45], although not in the context of breast cancer. A more surprising over-represented pathway was type II diabetes (with CACNA1C from our prioritized gene list). Some have reported that diabetes is associated with relatively poor breast cancer chemotherapy responses [46, 47]; therefore, a mechanistic link is plausible. Higher expression of CACNA1C, a subunit of the Cav1.2 voltage-gated calcium channel, has been associated with improved therapy response in B-cell lymphoma to a combination regimen including both doxorubicin and cyclophosphamide (closely related to the therapy in our study), although the authors concluded that CACNA1C impacted on response to the rituximab component of their combination [48].

The remaining candidate genes did not associate with survival in validation analyses and were not components of over-represented molecular pathways: ARAP2, FLG2, and DMBT1. ARAP2 is a GTPase-activating protein for the ADP-ribosylation factor family [49], but has not been assigned specific functions in cancer. Similarly, FLG2 (filaggrin 2) has not been implicated in cancer, but is involved with skin homeostasis through interactions with keratin [50]. Interestingly, filaggrin 2 is a member of the same protein family and is encoded in the same gene cluster as TCHH/trichohyalin described above [51], further implicating keratin dynamics in modulation of chemoresponse. Finally, DMBT1 is a secreted scavenger receptor [52], which is a potential tumour suppressor and reportedly increases sensitivity to the chemotherapeutic cisplatin [53].

In summary, we present evidence to implicate a novel list of genes in defining chemoresponse in breast cancer, and we propose these gene products as targets for chemosensitizing strategies or as predictive markers in order to improve outcomes for breast cancer patients.

## Electronic supplementary material

Below is the link to the electronic supplementary material.

Electronic supplementary material 1 (PDF 181 kb)

Electronic supplementary material 2 (XLSX 275 kb)

Electronic supplementary material 3 (XLSX 41 kb)
