# Supplementary material for: Identification of candidate mediators of chemoresponse in breast cancer through therapy-driven selection of somatic variants
Source: Breast Cancer Res Treat. 2020 Jul 30;183(3):607–16. doi: 10.1007/s10549-020-05836-7 (PMC7497675; doi:10.1007/s10549-020-05836-7)
Supplement: Supplementary file 1 — Electronic supplementary material 1 (PDF 181 kb) [file 10549_2020_5836_MOESM1_ESM.pdf]

Supplementary figure/tables

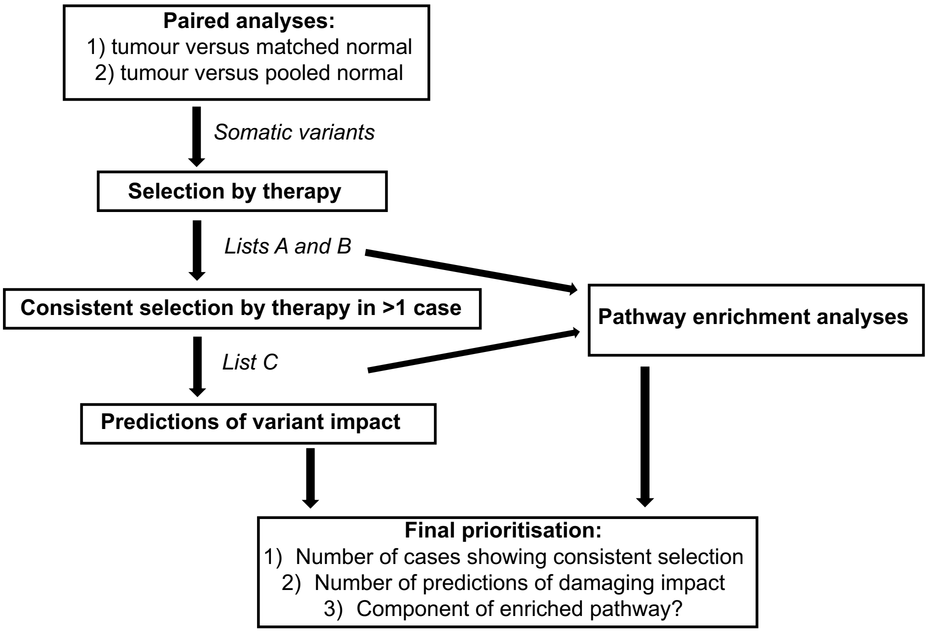

Figure S1. A flow-scheme demonstrating the method for identification of candidate regulators of chemoresponse, and the method for their prioritization.

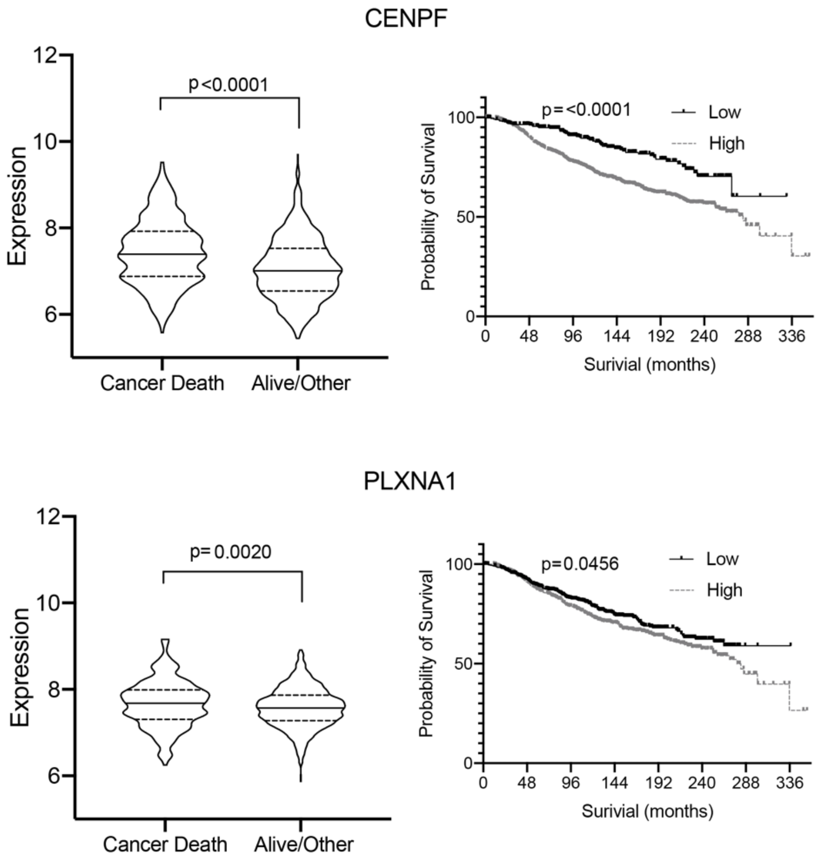

Figure S2. Expression of candidate genes correlated with breast cancer outcomes in estrogen receptor positive / HER2 negative cases. Expression levels of candidate genes in Table 2 were analysed for correlations with survival from breast cancer using the METABRIC dataset, by comparing the distribution of levels between patients who died of their cancer to those that did not using ‘violin’ plots (left of each pair), and by Kaplan-Meier analyses after expression was dichotomized using Receiver Operator Curve analyses into low and high groups (right of each pair). For violin plots, median and quartiles are shown (horizontal lines) and significance was tested using 2-tailed Mann–Whitney U tests. For Kaplan-Meier analyses, significance was tested using log rank tests. Significant correlations only are shown.

Excel-sheet

Table S1. Variants assigned to list A (selected against by chemotherapy) or list B (selected for by chemotherapy).

**List A (n=2078)**	**p-value**	**Gene matches**	**Total genes within pathway**
Collagen proteins	1.332E-11	22	44
Collagen chain trimerization	7.034E-11	22	47
Collagen biosynthesis /modifying	3.917E-09	25	70
Collagen formation	3.526E-08	28	93
ECM, glycoproteins, collagens proteoglycans	7.621E-08	56	275
Extracellular matrix organization	1.200E-07	56	298
Integrin signalling pathway	1.527E-06	37	167
Assembly of collagen fibrils and other multimeric structures	2.377E-06	19	60
Structural components of basement membranes	1.391E-05	14	40
Focal adhesion	9.509E-05	37	199
NCAM1 interactions	1.333E-04	12	37
Rap1 signaling pathway	1.392E-04	38	210
Diseases associated with protein O-glycosylation	1.509E-04	16	60
Diseases of glycosylation	1.883E-04	20	86
**List B (n=658)**	**p-value**	**Gene matches**	**Total genes within pathway**
Type II diabetes mellitus	3.610E-05	9	46
Integrin signaling pathway	4.034E-05	18	167
ECM, glycoproteins, collagens and proteoglycans	7.459E-05	24	275
Structural components of basement membranes	8.337E-05	8	40
**List C (n=132)**	**p-value**	**Gene matches**	**Total genes within pathway**
ECM, glycoproteins, collagens and proteoglycans	2.226E-06	11	275
Extracellular matrix and extracellular matrix-associated proteins	1.385E-04	18	1028
Structural components of basement membranes	1.465E-04	4	40
Collagen proteins	2.131E-04	4	44

Neurophilin interactions with VEGFR	2.675E-04	2	4
Collagen chain trimerization	2.758E-04	4	47
Genes encoding structural EMC glycoproteins	3.504E-04	7	196
Collagen formation	4.047E-04	5	93

Table S2. Genes showing therapy-driven selection are significantly enriched for functional groupings. Genes containing somatic variants showing selection by epirubicin/cyclophosphamide chemotherapy in breast cancers were identified in 6 individual cancers (lists A and B) or shared between at least 2 of 6 cancers (list C). Over-representation of functional groups was assessed using in silico analyses (ToppGene suite). Functional groups are listed in order of statistical significance (assessed as p-values, adjusted for multiple testing), including information on how many genes within the group under test were identified within each functional group (“Gene matches”), and how many genes in total were defined within that group in the ToppGene platform (“Total genes within pathway”).

Excel-sheet

Table S3. Variants in the 14 genes prioritized for further study.
